# Supplementary material for: Children’s Symptoms with a Febrile Illness and a Positive or Negative Test of SARS-CoV-2 during the Omicron Wave
Source: Children (Basel). 2023 Feb 22;10(3):419. doi: 10.3390/children10030419 (PMC10047075; doi:10.3390/children10030419)
Supplement: Supplementary file 1 [file children-10-00419-s001.zip › children-2201797-supplementary.pdf]

Supplementary Table S1. Symptomatic comparison of episodes with a positive and negative Corona saliva test during omicron wave

		Negative Corona Test		Positive Corona Test			
			N		N	p	t(3130)
**Temperature**	Mean (n)	38.7 (2497)	3132	38.6 (635)	3132	<0.001**	3.736
	SD	0.85		0.84			
	Median	38.7		38.6			
	Min	35.6		34.3			
	Max	41.8		41.1			
**Max. Temp.**	Mean (n)	38.9 (2497)	3132	38.9 (635)	3132	0.979	-0.027
	SD	0.92		0.93			
	Median	39.0		39.0			
	Min	35.6		34.3			
	Max	41.8		41.1			
		**% (n)**	**N**	**% (n)**	**N**	**p**	**Odds ratio**
**Fever (38.5°C)**		70.1%	2497	72.30% (459)	635	0.278	1.113 [0.917 – 1.351]
**Symptom**	**Any symptom**	82.5% (2080)	2521	80.9% (513)	634	0.349	0.899 [0.719 - 1.124]
	Cough	45.0% (1134)		60.7% (385)		<0.001**	1.891 [1.583 – 2.259]
	Constrained breathing	22.9% (578)		24.1% (153)		0.520	1.069 [0.872 – 1.312]
	Freezing/Chills	14.9% (376)		16.4% (104)		0.351	1.119 [0.883 – 1.419]
	Fatigue	61.2% (1541)		68.9% (437)		<0.001**	1.411 [1.171 – 1.700]
	Smell/taste disturbed	0.7% (17)		3.2% (20)		<0.001**	4.798 [2.498 – 9.214]
	Mucus	41.5% (1047)		39.1% (248)		0.269	0.905 [0.757 – 1.081]
	Tonsillitis	2.1% (54)		0.6% (4)		0.017*	0.290 [0.105 – 0.804]
	Joint swelling	0.2% (6)		0.3% (2)		0.730	1.326 [0.267 – 6.588]
	Teething	17.7% (447)		12.9% (82)		0.004*	0.689 [0.535 – 0.888]
	Something else	7.5% (188)		7.6% (48)		0.923	1.016 [0.731 – 1.413]
**Pain**	**Any pain symptom**	39.9% (811)	2035	33.0% (172)	521	0.004*	0.744 [0.607 – 0.911]
	Pain in the limbs	15.4% (314)		22.8% (119)		<0.001**	1.622 [1.280 – 2.057]
	Headache	17.9% (364)		21.1% (110)		0.091	1.229 [0.967 – 1.560]
	Sore throat	16.1% (328)		21.1% (110)		0.007*	1.393 [1.094 – 1.773]
	Pain in the ears	6.4% (131)		2.7% (14)		<0.001**	0.401 [0.229 – 0.702]
	Stomach ache	14.3% (290)		14.4% (75)		0.933	1.012 [0.769 – 1.331]
	Somewhere else	9.4% (192)		7.3% (38)		0.128	0.755 [0.526 – 1.085]
**Warning Sign**	**Any warning sign**	11.8% (291)	2466	9.7% (60)	616	0.151	0.807 [0.602 – 1.081]
	Touch sensitivity	3.8% (94)		6.8% (42)		<0.001**	1.846 [1.269 – 2.687]
	Shrill screaming	5.0% (124)		6.3% (39)		0.196	1.277 [0.881 – 1.850]
	Apathy	4.8% (118)		4.2% (26)		0.553	0.877 [0.568 – 1.354]
	Seems seriously sick	9.9% (244)		13.0% (80)		0.025*	1.359 [1.038 – 1.780]
**Neck stiffness**	Neck stiffness	2.8% (10)	356	0.9% (1)	107	0.470†	0.326 [0.041 – 2.579]
**Rash**	Rash	6.9% (172)	2498	3.3% (21)	635	<0.001**	0.463 [0.291 – 0.734]
**Diarrhea**	Diarrhea	16.5% (417)	2522	19.2% (123)	639	0.103	1.203 [0.963 – 1.504]
**Dehydration**	**Any sign of dehydration**	10.4% (258)	2472	8.1% (51)	626	0.088	0.761 [0.556 – 1.042]
	Dry mucous	1.9% (46)		3.0% (19)		0.070	1.651 [0.960 – 2.838]
	Dry Skin	1.0% (24)		1.4% (9)		0.313	1.488 [0.688 – 3.217]
	Tiredness	12.5% (310)		11.0% (69)		0.301	0.864 [0.655 – 1.140]
	Sunken eye sockets	1.6% (40)		2.6% (16)		0.119	1.595 [0.887 – 2.867]

	Less wet diapers	5.9% (147)	7.3% (46)	0.195	1.254 [0.890 – 1.768]
	Fontanelle is sunken	0.2% (5)	0.6% (4)	0.088†	3.173 [0.850 – 1.851]

Note: p-Value based on a X^2-Test with a significance level of 5%; *< 0.05; ** p < 0.001; †= p-value based on exact fisher test

Supplementary Table S2. Significant differences in symptoms between different age groups with a positive Corona saliva test during the omicron wave

	**0-2 years**	**3-5 years**	**6-17 years**			
	**% (n)**	**% (n)**	**% (n)**		**p**	**Odds ratio**
**Fever**	71.47% (268)	77.84% (144)	62.67% (47)	0-2 years vs. 3-5 years	-	-
				0-2 years vs. 6-17 years	-	-
				6-17 years vs. 3-5 years	0.013	2.092 [1.169 – 3.746]
**Symptoms**						
Cough	63.7% (237)	59.9% (109)	48.8% (39)	0-2 years vs. 3-5 years	-	-
				0-2 years vs. 6-17 years	0.014	0.542 [0.333 – 0.882]
				6-17 years vs. 3-5 years	-	-
Const. breathing	29.8% (111)	18.1 %(33)	11.3% (9)	0-2 years vs. 3-5 years	0.003	0.521 [0.336 – 0.807]
				0-2 years vs. 6-17 years	0.001	0.298 [0.144 - 0.617]
				6-17 years vs. 3-5 years	-	-
Freezing/Chills	9.14% (34)	19.23% (35)	43.75% (35)	0-2 years vs. 3-5 years	<0.001	2.367 [1.421 – 3.942]
				0-2 years vs. 6-17 years	<0.001	7.732 [4.394 – 13.607]
				6-17 years vs. 3-5 years	<0.001	0.306 [0.172 – 0.544]
Teething	21.24% (79)	1.10% (2)	1.25% (1)	0-2 years vs. 3-5 years	<0.001	0.041 [0.010 – 0.170]
				0-2 years vs. 6-17 years	0.003	0.047 [0.006 – 0.343]
				6-17 years vs. 3-5 years	-	-
Other symptoms	6.5% (24)	6.0% (11)	16.3% (13)	0-2 years vs. 3-5 years	-	-
				0-2 years vs. 6-17 years	0.005	2.813 [1.364 – 5.802]
				6-17 years vs. 3-5 years	0.011	0.332 [0.142 – 0.777]
**Pain symptoms**						
Any pain symp.	28.52% (77)	29.82% (51)	55.0% (44)	0-2 years vs. 3-5 years	-	-
				0-2 years vs. 6-17 years	<0.001	3.063 [1.833 – 5.120]
				6-17 years vs. 3-5 years	<0.001	0.348 [0.201 – 0.602]
Headache	11.11% (30)	17.54% (30)	62.5% (50)	0-2 years vs. 3-5 years	-	-
				0-2 years vs. 6-17 years	<0.001	13.333 [7.386 – 24.070]
				6-17 years vs. 3-5 years	<0.001	0.128 [0.070 – 0.233]
Sore throat	19.63% (53)	17.54% (30)	33.75% (27)	0-2 years vs. 3-5 years	-	-
				0-2 years vs. 6-17 years	0.009	2.086 [1.201 – 3.623]
				6-17 years vs. 3-5 years	0.005	0.418 [0.227 – 0.767]
Stomach ache	7.41% (29)	20.47% (35)	25% (20)	0-2 years vs. 3-5 years	<0.001	3.217 [1.787 – 5.790]
				0-2 years vs. 6-17 years	<0.001	4.167 [2.109 – 8.231]
				6-17 years vs. 3-5 years	-	-
**Warning signs**						
Any Warn. sign	13.46% (49)	4.60% (8)	3.85% (3)	0-2 years vs. 3-5 years	0.003	0.310 [0.143 – 0.670]
				0-2 years vs. 6-17 years	0.026	0.257 [0.078 – 0.847]
				6-17 years vs. 3-5 years	-	-
Shrill screaming	9.62% (35)	2.30% (4)	0% (0)	0-2 years vs. 3-5 years	0.005	0.221 [0.077 - 0.633]
				0-2 years vs. 6-17 years	-	-
				6-17 years vs. 3-5 years	-	-
Diarrhea	19.40% (73)	14.80% (27)	28.75% (23)	0-2 years vs. 3-5 years	-	-
				0-2 years vs. 6-17 years	-	-
				6-17 years vs. 3-5 years	0.009	0.432 [0.229 – 0.813]

Note: p-Value based on a X^2-Test with a significance level of 5%

Supplementary Table S3. Significant differences in symptoms with a positive and negative Corona saliva test within age groups during the omicron wave

	Negative Corona Test		Positive Corona Test			
**0-2 years**	**% (n)**	**N**	**% (n)**	**N**	**p ***	**Odds ratio [95% CI]**
Cough	42.6% (518)	1216	63.70% (237)	372	<0.001	2.366 [1.861 – 3.007]
Fatigue	60.8% (739)	1216	69.4% (258)	372	0.003	1.461 [1.139 – 1.873]
Smell/Taste disturbed	0.3% (4)	1216	2.4% (9)	372	<0.001†	7.512 [2.300 – 24.537]
Teething	33.6% (409)	1216	21.2% (79)	372	<0.001	0.532 [0.404 – 0.701]
Pain in the limbs	12.7% (102)	804	22.2% (60)	270	<0.001	1.966 [1.380 – 2.802]
Sore throat	12.1% (97)	804	19.6% (53)	270	0.002	1.780 [1.232 – 2.571]
Pain in the ears	6.1% (49)	804	2.6% (7)	270	0.030	0.410 [0.183 – 0.917]
Pain somewhere else	13.8% (111)	804	8.1% (22)	270	0.016	0.554 [0.343 – 0.895]
Seems seriously sick	9.9% (118)	1194	15.7% (57)	364	0.002	1.693 [1.204 – 2.380]
Rash	9.0% (108)	1206	4.2% (16)	377	0.004	0.451 [0.263 – 0.772]
**3-5 years**						
Cough	48.5% (486)	1002	59.9% (109)	182	0.005	1.585 [1.150 – 2.186]
Smell/Taste disturbed	0.9% (9)	1002	3.3% (6)	182	0.018†	3.761 [1.322 – 10.699]
Any pain symptom	37.8% (352)	931	29.8% (51)	171	0.047	0.699 [0.491 – 0.995]
Any warning sign	10.0% (98)	978	4.6% (8)	174	0.026	0.433 [0.207 – 0.907]
Touch sensitivity	3.5% (34)	978	8.0% (14)	174	0.007	2.429 [1.275 – 4.628]
Rash	5.2% (52)	995	1.1% (2)	178	0.011†	0.206 [0.050 – 0.854]
Sunken eye sockets	0.9% (9)	987	2.8% (5)	178	0.042	3.141 [1.040 – 9.483]
**6-17 years**						
Tiredness	16.3% (48)	295	6.3% (5)	79	0.028†	0.348 [0.134 – 0.905]
Freezing/Chills	27.1% (82)	303	43.8% (35)	80	0.004	2.096 [1.260 – 3.488]
Smell/Taste disturbed	1.3% (4)	303	6.3% (5)	80	0.022†	4.983 [1.306 – 19.011]
Diarrhea	15.9% (48)	302	28.7% (23)	80	0.010	2.135 [1.203 – 3.791]
Headache	50.0% (150)	300	62.5% (50)	80	0.048	1.667 [1.005 – 2.765]

Note: *=p-Values based on X^2-Test; †= p-Values based on the exact fisher Test
